# Exploring Molecular Genetics Research on Obesity in Malaysia: Protocol for a Scoping Review

**DOI:** 10.2196/60838

**Published:** 2024-12-30

**Authors:** Liyana Ahmad Zamri, Norhashimah Abu Seman, Nur Azlin Zainal Abidin, Siti Sarah Hamzah

**Affiliations:** 1 Endocrine and Metabolic Unit Nutrition, Metabolism and Cardiovascular Research Centre Institute for Medical Research, National Institutes of Health, Ministry of Health Malaysia Selangor Malaysia

**Keywords:** genetics, biomarkers, molecular, obesity, Malaysia, scoping review, protocol

## Abstract

**Background:**

Obesity presents a growing challenge to public health, and its intricate association with genetics continues to be a compelling field of study. In countries such as Malaysia, where diverse genetic backgrounds converge, exploring the molecular genetics of obesity is even more imperative.

**Objective:**

This scoping review aimed to explore the literature on molecular genetics of obesity in Malaysia. Specifically, we sought to characterize existing studies, identify the genetic determinants of obesity, and assess their association with obesity predisposition in the population.

**Methods:**

This scoping review followed the methodology of the Joanna Briggs Institute and used the PRISMA-ScR (Preferred Reporting Items for Systematic Reviews and Meta-Analyses Extension for Scoping Reviews) checklist as its guiding framework. Searches were conducted using electronic databases such as PubMed, ScienceDirect, and Scopus, filtering for human studies published until March 2024. Eligible studies included peer-reviewed articles on the Malaysian population irrespective of age or sex. This review excluded review articles, book chapters, non–peer-reviewed conference proceedings, gray literature, and preclinical studies, and the reference lists of the retrieved studies were manually examined to ensure thorough inclusion. The articles were subjected to a 2-stage screening process (title/abstract and full text) conducted by 2 reviewers to assess eligibility. Eligible articles were then extracted following a data extraction framework and organized into a charting table. Only studies investigating the genetics of obesity in Malaysian populations were included.

**Results:**

As of March 2024, our extensive search strategy has yielded 572 records. After removing 153 duplicates, 419 records were screened by title and abstract, resulting in 47 selected for full-text review. Of these, 34 were chosen for data extraction and detailed analysis. These studies predominantly involved participants from major ethnic groups (Malay, Chinese, and Indian) recruited from local health centers and university communities. The articles primarily explored the relationship between specific gene variants and obesity or obesity-related health parameters. This ongoing research is expected to be completed with a comprehensive scoping review by April 2025.

**Conclusions:**

This review provides valuable insights into the genetic determinants of obesity in Malaysia, despite limitations such as no quality appraisal being conducted for the included studies and the search strategy being restricted to selected databases, potentially omitting relevant studies. However, this review ensured reliability and reproducibility by adhering to the Joanna Briggs Institute and PRISMA-ScR guidelines. Ultimately, this study advances the understanding of local research and sets the foundation for future molecular genetic studies to improve obesity risk prediction and management in Malaysia’s multiethnic population.

**International Registered Report Identifier (IRRID):**

DERR1-10.2196/60838

## Introduction

Obesity presents a substantial public health challenge, exerting a significant global impact on economies and health care systems. The World Obesity Atlas 2023 predicts that over 51% of the global population will become obese or overweight within the next 12 years [1]. In this global crisis, Malaysia, an upper–middle-income country with a diverse population, ranks as the fattest nation in Southeast Asia [2]. Currently, more than half of Malaysian adults are overweight or obese [3], which increases the risk of diabetes, high blood pressure, and hypercholesterolemia [4,5]. These noncommunicable diseases incur an economic toll of approximately RM 8.91 billion (equivalent to US $2.01 billion), derived from productivity losses in the working-age population [6]. Hence, targeted interventions and comprehensive strategies are imperative to address the escalating obesity burden in Malaysia.

Genetic variation across populations significantly contributes to differences in gene expression phenotypes, which can potentially affect the prevalence of obesity. While general healthy eating guidelines can benefit individuals with poor dietary habits, a “one-size-fits-all” approach is often insufficient. Health disparities persist owing to gene-diet interactions, are influenced by genetic ancestry, and significantly affect how different individuals respond to the same dietary recommendations [7,8]. Additionally, individuals’ genetic makeup significantly influences how obesogenic environments contribute to obesity predisposition [9,10]. For instance, the *FTO* gene variant rs9939609 has a significant correlation with BMI in populations of European and African ancestry [11,12] and some Asian populations [13-15] but varies in other Asian populations [16]. Ethnic-specific risk factors have significant relevance in Malaysia, where distinct ethnicities exhibit varying predispositions to obesity, resulting in certain groups having a higher prevalence of body fat [17] and metabolic syndrome [18].

Molecular genetic investigations into obesity are notably scarce despite the significant health burden posed by the conditions in this country. A scoping review revealed a marked surge in obesity research conducted in Malaysia from 2008 to 2017 in response to the unprecedented prevalence of overweight and obesity in the country. However, only 12.3% of studies used experimental methods to investigate potential biomarkers, with genetic studies receiving minimal attention [19]. Moreover, the lack of a systematic compilation of existing research hampers efforts to effectively build upon previous work, exacerbating the challenge of designing new studies to address the research gap. Identifying the genetic determinants linked to obesity is crucial for developing targeted interventions to address the distinct challenges encountered by each group in combating obesity.

While lifestyle modifications for obesity have shown promise in reducing weight and improving cardiometabolic risk factors in the population [20-23], sustaining these changes over the long term remains a significant challenge [24,25]. Studying the genetic factors and metabolic pathways is crucial for the treatment and prevention of obesity. A recent study showed that integrating genomic data into a digital precision weight-loss program greatly enhances model accuracy and intervention effectiveness through personalized coaching based on genetic risk [26]. Additionally, genetic variants linked to abdominal obesity have been found to predict waist circumference regain after weight loss, emphasizing the role of genetics in long-term weight management [27]. Studies such as the OBEGEN study underscore the importance of integrating genetic testing with clinical data to enhance the predictability of weight loss outcomes following bariatric surgery [28]. These findings are corroborated by subsequent research [29,30]. This integrative approach will facilitate the development of personalized and cost-effective treatments for obesity.

Therefore, the primary purpose of this scoping review is to map the existing knowledge and findings and highlight gaps to guide future research. This scoping review aimed to explore published articles on molecular genetic research on obesity in the Malaysian context. We evaluated the published evidence regarding (1) study characteristics, including participant profiles (ie, sex and ethnicity) and aims/objectives; (2) key findings focusing on genetic determinants of obesity; and (3) the degree of association between the markers and obesity predisposition indicated by measures such as minor allele frequency or odds ratio values.

## Methods

### Study Design

The scoping review adheres to the most recent edition of the Joanna Briggs Institute Manual for Evidence Synthesis [31], using the framework established by Arksey and O’Malley [32] and subsequently enhanced by Levac et al [33]. This framework encompasses five key steps: (1) defining the research question; (2) identifying relevant studies; (3) selecting studies; (4) charting the data; and (5) compiling, summarizing, and presenting the findings. This method aims to comprehensively map the scope of research on a given topic, reveal existing knowledge, identify gaps, clarify known and unknown facets [33], and situate findings within policy and practical contexts [34]. The findings adhered to the PRISMA-ScR (Preferred Reporting Items for Systematic Reviews and Meta-Analyses Extension for Scoping Reviews) reporting guidelines to guarantee transparency and reproducibility [35]. The PRISMA-ScR checklist is provided in Multimedia Appendix 1.

This scoping review was registered in the Open Science Framework database (registration QYZKA) [36]. Citations were organized and duplicates were systematically removed using EndNote (Clarivate Analytics). A preliminary search was performed to identify previously published reviews on this topic. The search conducted on March 1, 2024, encompassed the following databases: MEDLINE (Ovid), Cochrane Library, and PROSPERO, using the keywords: “obesity” AND “genetic” AND “Malaysia.” No other systematic or scoping reviews on this topic, either published or registered, were discovered.

### Step 1: Identify the Research Question

Following the guidance of Levac et al [33], scoping review questions maintained a broad scope, while ensuring precise definitions of concepts to develop a robust search strategy. In alignment with these principles, the formulation of the research questions investigated in this review arose from discussions and consensus among reviewers. Therefore, the primary research question was as follows: What are the genetic determinants of obesity prevalent in the Malaysian population and how strongly are they related to obesity predisposition? Additionally, we aimed to determine the pathways through which these genetic factors contribute to obesity predisposition.

### Step 2: Identify Relevant Studies

The second phase of the scoping review focused on establishing criteria for selecting the research to be incorporated into the review. Despite the broad nature of a scoping review, these criteria guide the search process and aid in identifying relevant sources. The scoping review included peer-reviewed literature from the MEDLINE/PubMed, ScienceDirect, and Scopus electronic databases. The reviewers used an iterative process guided by Levac et al [33] to identify key search phrases. The search strategy shown in Table 1 involves generating subject headings and a list of keywords, incorporating Boolean operators (eg, AND and OR), adjacencies, and truncations. Medical Subject Headings terms were integrated and adjusted across different databases. The search strings for each database were finalized based on this exploratory scoping phase. The detailed search strategy and strings for all databases are provided in Multimedia Appendix 2.

**Table 1:** Example of search terms and their combinations in PubMed database.

Search number	Query
1	“gene*”[Title/Abstract] OR “polymorphism*”[Title/Abstract] OR “snp”[Title/Abstract] OR “genetic*”[Title/Abstract] OR “protein*”[Title/Abstract] OR “dna”[Title/Abstract] OR “rna”[Title/Abstract] OR “genetic variant”[Title/Abstract] OR “mutation*”[Title/Abstract] OR “variant*”[Title/Abstract] OR “GWAS”[Title/Abstract]
2	“malay*”[Title/Abstract] OR “malaysian chinese”[Title/Abstract] OR “malaysian indian”[Title/Abstract] OR “malaysia”[Title/Abstract]
3	“obes*”[Title/Abstract] OR “overweight”[Title/Abstract] OR “obesity”[Title/Abstract] OR “obes*”[MeSH^a^ Terms] OR “obesity”[MeSH Terms] OR “overweight”[MeSH Terms]
4	#1 AND #2 AND #3

^a^MeSH: Medical Subject Headings.

This scoping review encompasses studies involving all ethnicities in Malaysia, including Malay; Chinese; Indian; and non-Malay indigenous groups from Sabah and Sarawak, known as Bumiputera Sabah and Sarawak, as well as other indigenous communities. Articles published in peer-reviewed journals were considered, whereas systematic reviews, meta-analyses, scoping reviews, gray literature, PhD theses, abstracts, book chapters, perspectives, opinions, and commentaries were excluded. In addition, nonreviewed conference proceedings were omitted due to feasibility constraints and limited information.

Following the preliminary search, we refined our search strategy to enhance the identification of eligible studies. First, we expanded the search to include all studies up to March 2024 by removing restrictions on the publication year. This decision was based on the observation that limiting the search to the past 5 years yielded fewer relevant results. We included articles in both English and Malay for comprehensive coverage relevant to the Malaysian context. Furthermore, we incorporated additional keywords related to molecular genetics to improve the sensitivity of our search strategy. The inclusion and exclusion criteria are presented in Table 2.

**Table 2:** Inclusion and exclusion criteria.

Criteria	Inclusion	Exclusion
Studies to be assessed	Molecular research on obesity that explores genetic determinants	Nonmolecular obesity study
Literature	Peer-reviewed research paper	Gray literature, PhD theses, abstracts, systematic reviews, meta-analyses, scoping reviews, book chapters, perspectives, opinions, commentaries, and nonreviewed conference proceedings.
Species	Human/clinical studies	Preclinical studies encompass both in vivo and in vitro research
Population	Study conducted among Malaysians (Malay, Chinese, Indian, Bumiputera Sabah and Sarawak and indigenous people), of all ages and sexes	Non-Malaysian population
Language	Articles published in English and Malay	Non-English or non-Malay articles
Publication date	All until March 2024	—^a^

^a^Not applicable.

### Step 3: Study Selection

After the search, all identified articles were retrieved from each database and imported into the reference management software EndNote (version X9; Clarivate Analytics) in RIS/nbib file format. The team manually scanned all the included studies to locate other relevant studies. The articles in Endnote were screened for duplication and eliminated before being imported into Microsoft Excel spreadsheets.

Two independent reviewers screened the selected titles and abstracts based on the eligibility outlined in the inclusion and exclusion criteria and assessed whether the content addressed the research questions. The next step consisted of obtaining the full texts of the selected and identified articles and distributing them equally among the team members. Two reviewers independently screened the entire texts of the articles and were required to justify the reasons for their exclusion. Any disagreements among the reviewers during the selection process were resolved through discussion or with the involvement of a third reviewer, until a complete consensus was reached. The scoping review reported and documented the reasons for omitting sources during the full-text evaluation. The search results will be incorporated into the planned scoping review guided by the PRISMA-ScR flow diagram and checklist [25].

### Step 4: Extract and Chart the Data

The team initially reviewed several articles to ensure a thorough understanding of the conceptual framework and identified essential data to address the research questions. Subsequently, a data collection form was developed in accordance with the data extraction framework (Table 3) to assess full articles that met the eligibility criteria. Prior to implementation, all reviewers examined and pretested the form to ensure its effectiveness in capturing pertinent information. Data extraction was conducted in duplicate, and 2 reviewers independently extracted data from all eligible articles. Discrepancies between the extracted data were carefully examined to ensure their accuracy and consistency. Based on feedback from team members, the data extraction form was revised before final adoption. The compiled data were organized into a single Microsoft Excel document for validation and coding. Finally, the reviewers agreed upon a standardized data extraction method that encompassed all relevant information.

**Table 3:** Data extraction framework.

Main category and subcategory			Description
**Bibliographic information**			
	Authors	—^a^	
	Title	—	
	Year of publication	—	
	Journal name	—	
**Objectives of the study**			
	—	Indicate the objectives or aims of the study	
**Study population**			
	Target population	Specify if the study (1) targets individuals within a subpopulation or (2) broad population	
	By BMI	Specify the distribution of the study population by BMI	
	By gender	Specify if the study focuses on a specific gender, particularly girls, boys, and older people, or indicate the distribution of study participants by gender	
	By ethnic background	Specify if the study targets certain ethnic groups	
	By sample size	Indicate the sample size of the study population and each study group (eg, control and treatment if applicable)	
**Results and key findings**			
	Main findings	Summary of main findings	
	Genetic determinants	Identify the genetic factors influencing obesity predisposition, including variants, mutations, and gene expression patterns	
	Association with obesity	Indicate the degree of genetic association with obesity, including measures such as minor allele frequency (MAF) or odd ratio (OR) (if applicable)	
**Discussion and conclusion**			
	Conclusion	Indicate the conclusion stated in the study	
	Study implications	Implications for practice, policy, or further research	
	Study limitations	Limitations/challenges of the study or approach as stated by authors	

^a^Not applicable.

### Step 5: Collate, Summarize, and Report the Results

The proposed scoping review identifies the available information on the human genetics of obesity in the Malaysian context, summarizes the findings, and contextualizes the research questions. The charted evidence will be used to identify knowledge gaps and make recommendations for future research directions and needs in this field. A narrative approach will be used to report the findings, with results presented in aggregate as appropriate, and patterns and trends (if identified) illustrated in visual formats (eg, tables, figures, and charts).

## Results

The systematic scoping review protocol was initiated in February 2023. This study was conducted without any external funding. Preliminary data collection commenced in March 2023 and was updated in March 2024, resulting in 572 results from the electronic database searches. Following the removal of duplicates (n=153), 419 records were screened by title and abstract, resulting in 47 records selected for full-text review. Of these, 34 were retained for data extraction and analysis. A PRISMA flow diagram is presented in Multimedia Appendix 3. Most of the studies primarily focused on major ethnic groups, such as Malay, Chinese, or Indian, who were recruited from local health centers and university communities. These studies investigated the association between specific gene variants and obesity or obesity-related health parameters. The final results will be submitted for a systematic scoping review in April 2025.

## Discussion

The growing obesity crisis poses significant challenges to public health and the economy, with Malaysia being one of the most affected countries. Half of the adult population is currently overweight or obese, accompanied by an increase in noncommunicable diseases [3]. While environmental factors contribute to this surge, obesity results from a complex interplay between these factors and genetic predispositions, critically influencing individual susceptibility to the “obesogenic” environment.

The scope of this review specifically encompasses human or clinical studies while excluding preclinical investigations such as in vivo or in vitro studies. Prioritizing human or clinical studies on genetic obesity not only enhances our understanding of how genetic factors impact disease risk, treatment responses, and overall well-being but also has practical implications for personalized health care [37-39]. Despite the proven success of personalized medicine in guiding the prevention, diagnosis, and treatment of conditions, such as cancer, using genetic and phenotypic information, its implementation in addressing obesity remains limited. Moreover, this review was limited to research conducted in Malaysia, including studies across various ethnic groups and across all segments of the population, including both sexes.

This review synthesizes research from diverse ethnic backgrounds in Malaysia to identify knowledge gaps and prioritize future research questions, shaping the path for future studies. Revealing the trends and characteristics of genetic studies on obesity in Malaysia will guide researchers toward areas that are likely to yield new insights. This streamlined approach has expedited advancements in elucidating the molecular genetic basis of human obesity. Additionally, mapping research in this field can inform policy decisions on obesity and public health, aiding resource allocation and intervention design. The outcomes of this review will be shared through publications and presentations at conferences, enriching the scientific community’s understanding of the genetic factors that influence obesity in Malaysia. However, this scoping review has some limitations. First, we did not perform a quality appraisal of the included studies. Furthermore, the search strategy was limited to the selected databases, which may have led to the omission of relevant studies. Despite these limitations, this review adhered to the stringent guidelines of the Joanna Briggs Institute and PRISMA-ScR, ensuring its reliability and reproducibility.

This review aims to comprehensively explore and consolidate the existing literature on the molecular genetics of obesity in Malaysia. The discussion will analyze the findings within the framework of the study attributes and primary outcomes, focusing on genetic determinants and their association with obesity susceptibility. Furthermore, our study seeks to explore the identified genes and their corresponding pathways that influence the progression of obesity using a comprehensive integrative analytical approach. This approach offers a deeper understanding of the protein-protein interaction network, potentially providing valuable insights into the complex biological mechanisms underlying obesity.

## Appendix

Multimedia Appendix 1 PRISMA-P (Preferred Reporting Items for Systematic Review and Meta-Analysis Protocols) checklist.

Multimedia Appendix 2 Detailed search strategies for each database used in the scoping review.

Multimedia Appendix 3 PRISMA-ScR (Preferred Reporting Items for Systematic Reviews and Meta-Analyses Extension for Scoping Reviews) flow diagram.

## Abbreviations

PRISMA: Preferred Reporting Items for Systematic Reviews and Meta-Analyses
PRISMA-ScR: Preferred Reporting Items for Systematic Reviews and Meta-Analyses Extension for Scoping Reviews

## Data Availability

The datasets generated or analyzed during this study are available from the corresponding author on reasonable request.
