# Supplementary material for: Exploring Molecular Genetics Research on Obesity in Malaysia: Protocol for a Scoping Review
Source: JMIR Res Protoc. 2024 Dec 30;13:e60838. doi: 10.2196/60838 (PMC11729775; doi:10.2196/60838)
Supplement: Multimedia Appendix 2 [file resprot_v13i1e60838_app2.pdf]

Search conducted on March 5, 2024.

Database	Search	Query	Records retrieved
PubMed[a] (NLM)	#1	"gene*"[Title/Abstract] OR "polymorphism*"[Title/Abstract] OR "snp"[Title/Abstract] OR "genetic*"[Title/Abstract] OR "protein*"[Title/Abstract] OR "dna"[Title/Abstract] OR "rna"[Title/Abstract] OR "genetic variant"[Title/Abstract] OR "mutation*"[Title/Abstract] OR "variant*"[Title/Abstract] OR "GWAS"[Title/Abstract]	9,817,486
	#2	"malay*"[Title/Abstract] OR "malaysian chinese"[Title/Abstract] OR "malaysian indian"[Title/Abstract] OR "malaysia"[Title/Abstract]	35,486
	#3	"obes*"[Title/Abstract] OR "overweight"[Title/Abstract] OR "obesity"[Title/Abstract] OR "obes*"[MeSH Terms] OR "obesity"[MeSH Terms] OR "overweight"[MeSH Terms]	492,040
	#4	#1 AND #2 AND #3	193
[a] Limited to English, Malay, humans and from 1000/01/01 – 05/03/2024			
SCOPUS[b]	#1	(TITLE-ABS-KEY (malay*) OR TITLE-ABS-KEY ("malaysian chinese") OR TITLE-ABS-KEY ("malaysian indian") OR TITLE-ABS-KEY (malaysia))	169,829
	#2	(TITLE-ABS-KEY (obes*) OR TITLE-ABS-KEY (overweight) OR TITLE-ABS-KEY (obesity))	700,998
	#3	(TITLE-ABS-KEY (gene*) OR TITLE-ABS-KEY (polymorphism*) OR TITLE-ABS-KEY (snp*) OR TITLE-ABS-KEY (genetic*) OR TITLE-ABS-KEY (protein) OR TITLE-ABS-KEY (rna) OR TITLE-ABS-KEY (dna) OR TITLE-ABS-KEY (mutation*) OR TITLE-ABS-KEY (variant*) OR TITLE-ABS-KEY ("genetic variant") OR TITLE-ABS-KEY (gwas))	23,503,898
	#4	#1 AND #2 AND #3	514
	#5	((TITLE-ABS-KEY(gene*) OR TITLE-ABS-KEY(polymorphism*) OR TITLE-ABS-KEY(SNP*) OR TITLE-ABS-KEY(genetic*) OR TITLE-ABS-KEY(protein) OR TITLE-ABS-KEY(RNA) OR TITLE-ABS-KEY(DNA) OR TITLE-ABS-KEY(mutation*) OR TITLE-ABS-KEY(variant*) OR TITLE-ABS-KEY("genetic variant") OR TITLE-ABS-KEY(GWAS))) AND ((TITLE-ABS-KEY(obes*) OR	341

		TITLE-ABS-KEY(overweight) OR TITLE-ABS-KEY(obesity))) AND ((TITLE-ABS-KEY(malay*) OR TITLE-ABS-KEY("Malaysian Chinese") OR TITLE-ABS-KEY("Malaysian Indian") OR TITLE-ABS-KEY(malaysia))) AND (LIMIT-TO (DOCTYPE,"ar")) AND (LIMIT-TO (AFFILCOUNTRY,"Malaysia")) AND (LIMIT-TO (SRCTYPE,"j")) AND (LIMIT-TO (LANGUAGE,"English"))	
[b] Limited to English, Malaysia, research articles, journal			
**Science Direct[c]**	#1	(obese OR overweight OR obesity) AND (malay OR "malaysian chinese" OR "malaysian indian" OR Malaysia)	137
	#2	(gene OR polymorphism OR protein OR mutation OR variant) AND (malay OR "malaysian chinese" OR "malaysian indian" OR Malaysia)	859
		Combined the articles from search #1 and #2 in EndNote, filter was applied for "gene" and "obesity" to refine the results	38
[c] Search #1 and #2 limited to research articles, English, year 1000 - 2024			
