# Supplementary figures and images for: Exploring Molecular Genetics Research on Obesity in Malaysia: Protocol for a Scoping Review
Source: JMIR Res Protoc. 2024 Dec 30;13:e60838. doi: 10.2196/60838 (PMC11729775; doi:10.2196/60838)

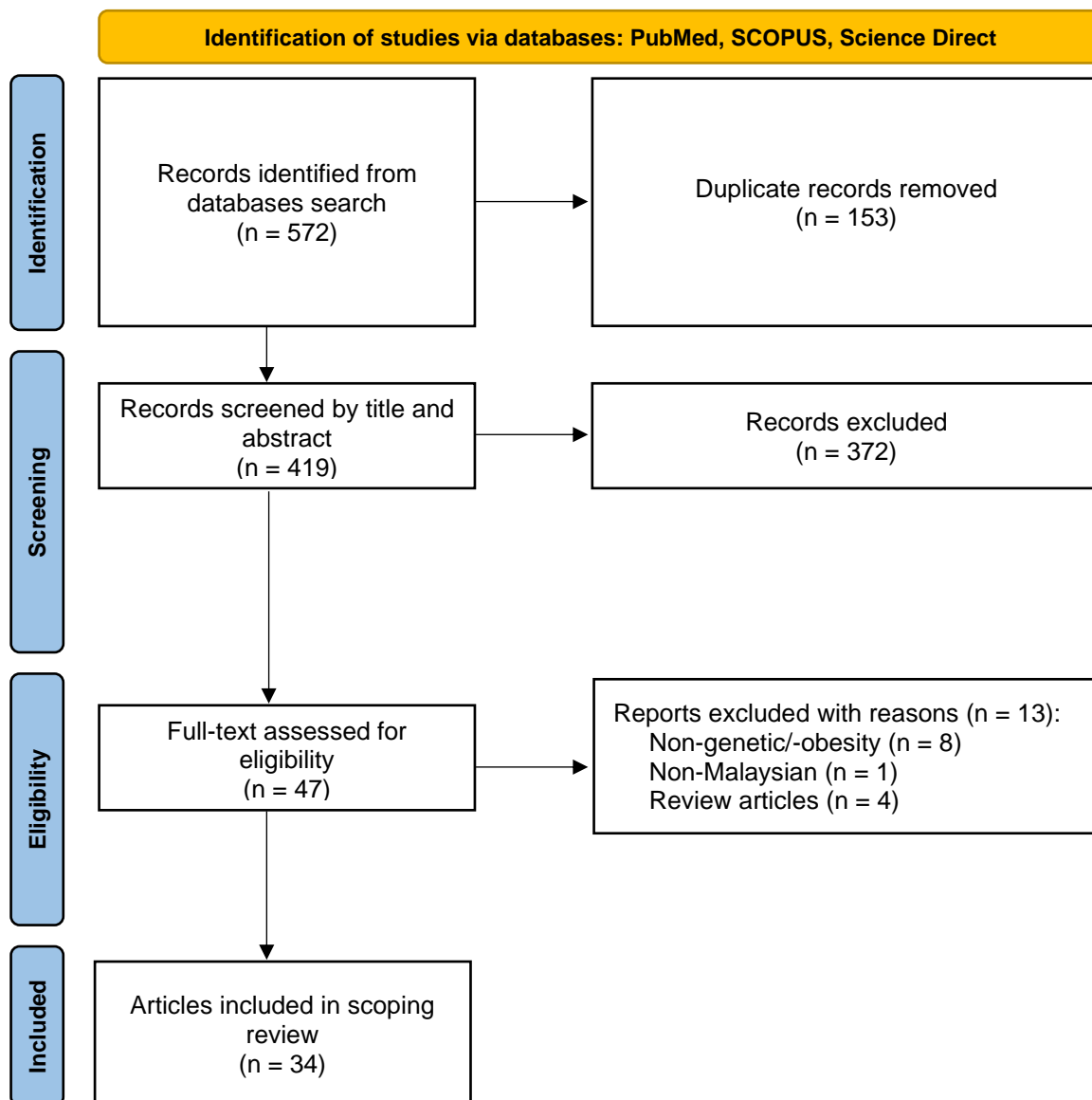

Supplement: Multimedia Appendix 3 [file resprot_v13i1e60838_app3.pdf]
